# Professional Morality in 1831

**Published:** 1831-04-01

**Authors:** 


PROFESSIONAL MORALITY IN 1831. 8vo. pp. 80, Wilson, March, 1831.

WE question the wisdom or the utility of this pamphlet, though we acknowledge that it is well written, and satirizes quackery in strong language. But quackery will flourish to the end of time — at all events, to the end of our time, in the present generation. While there are fools to believe the assertions of charlatans, there will be always abundance of the latter to fleece the public and enrich themselves, no matter what be the consequences of their empiricism. But when regular practitioners appeal to the public against the unprincipled empirics, the public are illiberal enough to suspect their motives, and attribute their hostility to jealousy. On this account we distrust the policy of public appeals against charlatanism. We have another objection to the present publication, namely, its reflections on the medical profession generally and specially—particularly the latter. On reflection, the author will be inclined to omit, in future, such passages as the following.

" Look at much of the GENERAL PRACTITIONER's conduct in the present day, and say, is it not, in its spirit and its objects, sheer quackery? Look at the thousand plans by which he endeavours to puff himself into notoriety. See the manner in which he manœuvres his course into new families — analyze the spirit with which he labels his draughts and makes out his bills—watch his anxiety to dispose of so much medicine in so many hours — question him on the seat or nature of nine-tenths of the diseases he attends, and of the philosophy of the treatment which he is pursuing for their removal—and, if my censures prove too severe, I shall be most happy in finding that I have calumniated those whom it is the object of these remarks to elevate beyond the mere art and grocery of their profession."

Now, while we must admit that there are too many individuals in every class of the profession, whose necessities or inclinations tempt to practise trickery and quackery in their avocations, it is not liberal to stigmatise one particular branch in such terms as the above passage conveys.

After these few remarks on the pamphlet before us, it is hardly worth while to contradict a general but most unfounded rumour that the Editor of this Journal is its author. He never wrote a line of it — nor has he the slightest knowledge of the writer.

